# Depression and cognition mediated rapid eye movement sleep behavior disorder to improve the activities of daily living in Parkinson’s disease patients

**DOI:** 10.3389/fpsyt.2026.1776297

**Published:** 2026-03-20

**Authors:** Jing Cui, Jiaqi Zhang, Yang Cheng, Durong Chen, Yao Qin, Xiao Lu, Hongmei Yu

**Affiliations:** 1 Third Hospital of Shanxi Medical University, Shanxi Bethune Hospital, Shanxi Academy of Medical Sciences, Tongji Shanxi Hospital, Taiyuan, Shanxi, China; 2 Department of Health Statistics, School of Public Health, Shanxi Medical University, Taiyuan, Shanxi, China; 3 Shanxi Provincial Key Laboratory of Biomedical Data and Statistics, Taiyuan, Shanxi, China

**Keywords:** causal mediation, cognitive function, depression, longitudinal mediation analysis, Parkinson’s disease, rapid eye movement sleep behavior disorder

## Abstract

**Introduction:**

Rapid eye movement sleep behavior disorder (RBD) aggravates cognitive impairment and depression in patients with Parkinson’s disease (PD) and significantly compromises their activities of daily living (ADL). This study aimed to explore the potential mediation roles of depression and cognitive impairment between RBD and ADL in PD patients.

**Methods:**

Data from the Parkinson's Progression Markers Initiative (PPMI) database comprised 337 patients. RBD, ADL, depression, and cognitive function were assessed with validated rating scales. Bayesian dynamic mediation analysis was employed to evaluate the separate mediating effects of depression and cognition in mediating RBD and ADL with the time variable (9 times) standardized to the interval [0, 1] via min–max normalization. Multiple time-varying causal mediation analysis was used to assess the role of depression and cognition together in mediating RBD and ADL.

**Results:**

At the second follow-up (0.25), the fourth follow-up (0.50), and the sixth follow-up (0.75), the Bayesian dynamic indirect mediating effect of depression between RBD and ADL were 0.058 (95%*CI* 0.039-0.077), 0.065 (95%*CI* 0.046-0.084), 0.082 (95%*CI* 0.061-0.103). The corresponding time-function curve revealed that the mediating effect of depression gradually increased with time. At the 0.25, 0.50, 0.75 standardized time points, the Bayesian dynamic indirect mediating effect of cognition between RBD and ADL were 0.062 (95%*CI* 0.038-0.086), 0.087 (95%*CI* 0.044-0.130), 0.136 (95%*CI* 0.096-0.176), and its time-function curve likewise indicated an increasing trend over time. The joint mediating analysis of the bidirectional decomposition method of the multiple time-varying causal mediation model confirmed that the joint mediating effect of depression and cognition between RBD and ADL was 7.42 (95%*CI* 4.48-10.36).

**Discussion:**

This study demonstrates that depression and cognition can work independently and together, and that reducing depressive symptoms or improving cognition can mediate the reduction of RBD to improve the ADL of PD patients. Notably, this mediating effect gradually intensifies as the duration of the disease progresses. These findings highlight that for PD patients, preventing emotional depression and cognitive decline in the early stage of the disease may reduce the damage caused by RBD.

## 1 Introduction

Non-motor symptoms (NMS) of Parkinson’s disease (PD) lead to a decrease in patients’ quality of life, increased disability, and an increase in the overall burden of the disease (1). Rapid eye movement sleep behavior disorder (RBD) is one of the most NMS in PD patients (2) and is closely related to multiple NMS (3, 4), especially exacerbating psychiatric symptoms such as depression and cognitive impairment (5).

RBD directly impairs PD patients’ activities of daily living (ADL) through sleep fragmentation and nocturnal behavior abnormalities. However, RBD not only has a direct effect on ADL, but may also indirectly affect ADL through depression. Because RBD often coexists with depression, and there may be common neurobiological mechanisms underlying both conditions, such as dysfunction in the brainstem’s monoaminergic system (6, 7). At the same time, depression reduces ADLs in PD patients (8). This suggests that depression may act as a mediating variable between RBD and ADL in PD patients. In addition, there is a bidirectional association between depression and cognitive function. Depression can accelerate cognitive impairment through elevated cortisol (9), which in turn exacerbates depression due to frustration and social withdrawal. When depression and cognitive impairment coexist, the damage to ADL is superimposed (10). Luo et al. (11) investigated the mediating role of PD severity in the relationship between sleep quality and ADL in PD patients, as well as the moderating effect of cognition on this association, confirming that cognitive function plays a regulatory role in PD patients. Therefore, RBD may also indirectly affect ADL through cognition. Cortical and subcortical gray matter abnormalities in patients with RBD are associated with their cognitive status (12), while cognitive decline has an impact on ADL in PD patients (13). RBD may not only directly affect ADL, but may also indirectly weaken ADL in patients by exacerbating depression or cognitive impairment. However, there is still a lack of mediation analysis based on longitudinal design, and most of the existing mediation studies focus on a single mediating variable. Therefore, further longitudinal studies are needed to explore the causal pathways among depression, cognitive function, RBD, and ADL in this population, which is of great significance for understanding the NMS mechanism of PD, identifying high-risk groups with impaired ADL, and developing early intervention strategies.

Based on the causal mediation model of the counterfactual framework, this study aims to demonstrate the independent causal mediating and joint mediating effects of depression and cognition in RBD and ADL in PD patients, and further quantify the magnitude of these mediating effects, so as to provide causal evidence for understanding the complex network of NMS in PD patients.

## 2 Materials and methods

### 2.1 Study population

**Figure 1:**
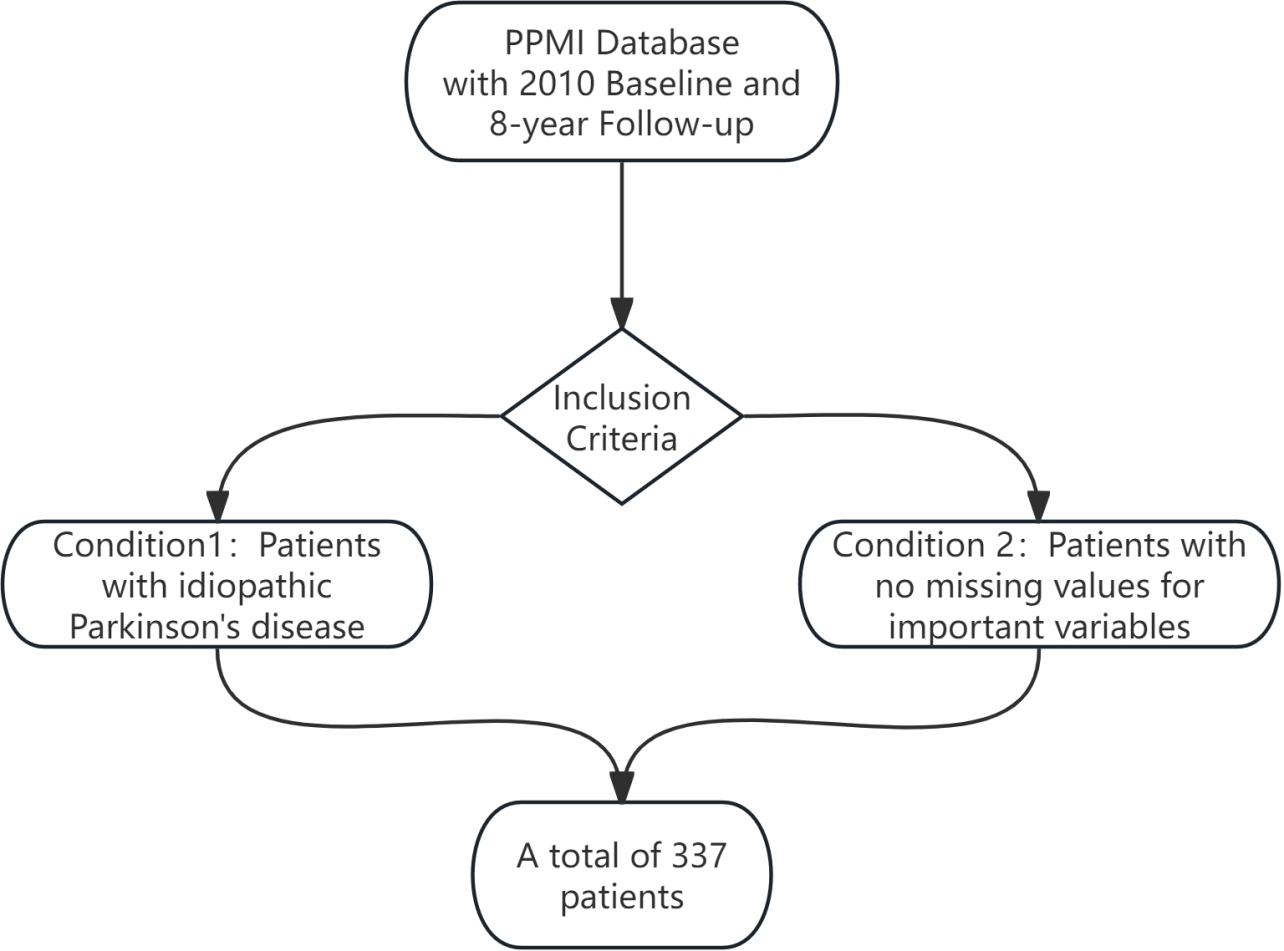
Flow diagram for participants of PPMI.

Data used in the preparation of this article were obtained from the Parkinson’s Progression Markers Initiative (PPMI) database (https://www.ppmi-info.org/access-data-specimens/download-data), RRID: SCR_006431. For up-to-date information on the study, visit http://www.ppmi-info.org. This longitudinal study utilized data from a baseline assessment in 2010 followed by 8 years of annual follow-ups (total 9 timepoints, 12-month intervals), comprising a final cohort of 337 patients (see **Figure 1**). The original PPMI study protocol had been approved by the Institutional Review Committee of all participating centers, and all participants had signed written informed consent. As a secondary analytical study of de-identified data, it was confirmed that it met the criteria for exemption from informed consent. This study adhered to the terms of PPMI’s Data Use Agreement throughout the study.

### 2.2 Assessment of primary variables

#### 2.2.1 Evaluation of RBD

The rapid eye movement sleep behavior disorder screening questionnaire (RBDSQ) was used for assessment. There are 10 items in total, and the score ranges from 0–13 points, and the higher the score, the more severe the symptoms of RBD (14). As reported by Karin Stiasny-Kolster et al. (15), the RBDSQ showed good internal consistency, with a Cronbach’s α of 0.885.

#### 2.2.2 Evaluation of ADL

Assessed using the movement disorder society-sponsored revision of the Unified Parkinson’s Disease Rating Scale part two (UPDRS II) (16). It covers 13 aspects such as verbal communication, saliva control and dripping, with a total score of 52, and the closer the score is to 52, the more severely the PD patients are limited in ADL.

#### 2.2.3 Evaluation of depression

The Geriatric Depression Scale-15 (GDS-15) was used to assess the severity of depression in PD patients. It contains 15 items, with a score range of 0-15. A score of ≥5 indicates mild depression, while a score of ≥ 10 indicates moderate to severe depression (17).

#### 2.2.4 Evaluation of cognitive function

The Montreal Cognitive Assessment (MoCA) was employed to evaluate cognitive function. It covers 8 cognitive domains across 11 items, with total scores ranging from 0-30. A score of ≥26 indicates normal cognitive level (18).

### 2.3 Assessment of covariates

Based on previous studies, the covariates included in this study were sex (male or female), age (<56, 56–65 or >65), years of education (<13, 13–23 or >23), race (White, Black, Asian or other), family history (first-degree family history, non-first-degree family history or no family history), years of disease duration (<5, 5–10 or >10).

### 2.4 Statistical analysis

Descriptive statistics reported the baseline characteristics of participants, with categorical variables were summarized as counts (percentage). The ADL values of baseline data and 8 follow-up data were statistically described by mean ± standard deviation (SD). Two-sample t-tests and one-way ANOVA were employed to examine differences in ADL scores among PD patients with distinct pathological features. Pearson correlation analysis was used to assess the bivariate relationships between RBD, depression, cognition, and ADL.

### 2.5 Mediation analysis

Mediation analysis is a research method used to investigate whether the independent variable X exerts an effect on the dependent variable Y through a mediating variable M (19).

We employed a Bayesian dynamic mediation model for the following reasons. Firstly, it captures time-varying effects, allowing the coefficients of the mediation pathways to vary dynamically over time. This better aligns with real-world developmental processes: *α(t)* captures the temporal effect of X on the mediator M, and *β(t)* measures the time-varying effect of M on Y, adjusted for X (20). Secondly, it accommodates complex data structures, effectively handling repeated measurements and individual differences through the incorporation of random effects and hierarchical modeling. The time variable for the 9-timepoint data was subjected to min–max normalization, linearly mapping it to the interval [0, 1]. Based on this consideration, the Bayesian dynamic mediation model was constructed. Covariates sex, age, education, race, family history, and disease duration were all naturally integrated into the construction process of the dynamic pathway itself. The mediating effects of depression and cognition in the relationship between RBD and ADL were evaluated separately. The posterior parameter trajectories of the Bayesian dynamic mediation model were used to assess the accuracy of the model. After 10,000 iterations, the Markov chains reached a stable state, indicating that the convergence of the model parameters was reasonable.

Multiple time-varying causal mediation are introduced into the longitudinal causal mediation analysis based on the mediated g-formula. When estimating the effects at each timepoint, we controlled for all covariates up to that timepoint to block the confounding path. We implemented multivariate time-varying causal mediation via two approaches (21): (i) Two-way decomposition: Mediators jointly define a single indirect effect (*vs*. direct effect); (ii) Path-specific decomposition: Quantifies individual mediators’ contributions to indirect effects, contingent on predefined causal structures and stronger identifiability assumptions. The *ξ* parameter represents the residual between the Total Effect (TE) and the total effect estimated by the model.

Statistical descriptions and correlation analyses were performed with SPSS 26.0. The Bayesian dynamic mediation models were constructed using R 4.3.3, and the multiple time-varying causal mediation model was implemented using “gfoRmula” package. The Monte Carlo simulation was used to perform *post-hoc* power analysis to estimate statistical power with “lavaan” package in R 4.4.1 (22). A two-tailed *P* < 0.05 was considered statistically significant in all tests.

## 3 Results

### 3.1 Basic information about the subject of the study

A total of 337 PD patients were included in this study, of which 65.28% were male and 74.78% were older than 65 years old. Most patients were without family history of PD, had disease duration for more than 10 years, and were Caucasians. The results indicated that there was no significant difference in ADL scores between demographic data groups at baseline, as detailed in **Table 1**.

**Table 1:** The ADL scores of study subjects.

Variable	N (%)	Mean ± SD	*t/F*	*P*-value
Sex				
Female	117 (34.72)	3.44 ± 3.57	3.027	0.083
Male	220 (65.28)	4.30 ± 4.12		
Age (years)				
<56	11 (3.26)	5.45 ± 5.75	1.831	0.162
56~65	74 (21.96)	4.54 ± 3.69		
>65	252 (74.78)	3.78 ± 3.92		
Education (years)				
<13	75 (22.26)	2.75 ± 3.05	5.168	0.106
13~23	258 (76.56)	4.34 ± 4.12		
>23	4 (1.18)	5.50 ± 4.12		
Race				
Whites	324 (96.14)	4.04 ± 3.99	0.890	0.447
Blacks	2 (0.59)	0.50 ± 0.71		
Asian	5 (1.48)	4.80 ± 2.68		
other	6 (1.79)	2.50 ± 2.07		
Family history				
First-degree	73 (21.66)	3.53 ± 3.83	0.695	0.500
Non-first-degree	57 (16.91)	4.00 ± 3.73		
No	207 (61.43)	4.17 ± 4.06		
Disease duration(years)				
<5	54 (16.02)	4.02 ± 3.36	0.159	0.853
5~10	82 (24.33)	4.21 ± 4.56		
>10	201 (59.67)	3.92 ± 3.85		

Pearson correlation analysis was used to examine correlations between RBD, depression, cognition, and ADL, and the results were shown in **Table 2**, showing a positive correlation between the RBD, depression, and ADL (*P*<0.01) and a negative correlation between the cognition and RBD, depression, and ADL (*P*<0.01). The correlation coefficient matrix heat map of each key variable was shown in **Supplementary Figure 1**.

**Table 2:** Correlation analysis of RBD, depression, cognition and ADL.

Variables	RBD	Depression	Cognitive	ADL
RBD	1			
Depression	0.179**	1		
Cognitive	-0.113**	-0.173**	1	
ADL	0.340**	0.258**	-0.183**	1

*P < 0.05, **P < 0.01, ***P < 0.001.

### 3.2 Depression as a mediator

Using RBD as the independent variable, ADL as the dependent variable, and depression as the mediating variable, we constructed a Bayesian dynamic mediation model. We first explored the mediating effects at different time points, three representative time points, the second follow-up (0.25), the fourth follow-up (0.50), and the sixth follow-up (0.75), were selected to present the results of their indirect mediating effects test (**Table 3**). The indirect effects of cognition showed an increasing trend over time, increased from 0.058 to 0.082, a growth rate of approximately 41%. The standard deviations of the indirect effects were 0.028, 0.030, and 0.053, respectively, with a slight increase over time. At time points 0.25, 0.50, and 0.75, the 95%*CI* for the mediation effects were 0.039-0.077, 0.046-0.084, and 0.061-0.103.

**Table 3:** Testing the mediating effect in a Bayesian mediation model with depression as the mediating variable.

Time point	Indirect effect	Standard deviation	95%*CI*
0.25	0.058	0.028	(0.039, 0.077)
0.50	0.065	0.030	(0.046, 0.084)
0.75	0.082	0.053	(0.061, 0.103)

**Figure 2:**
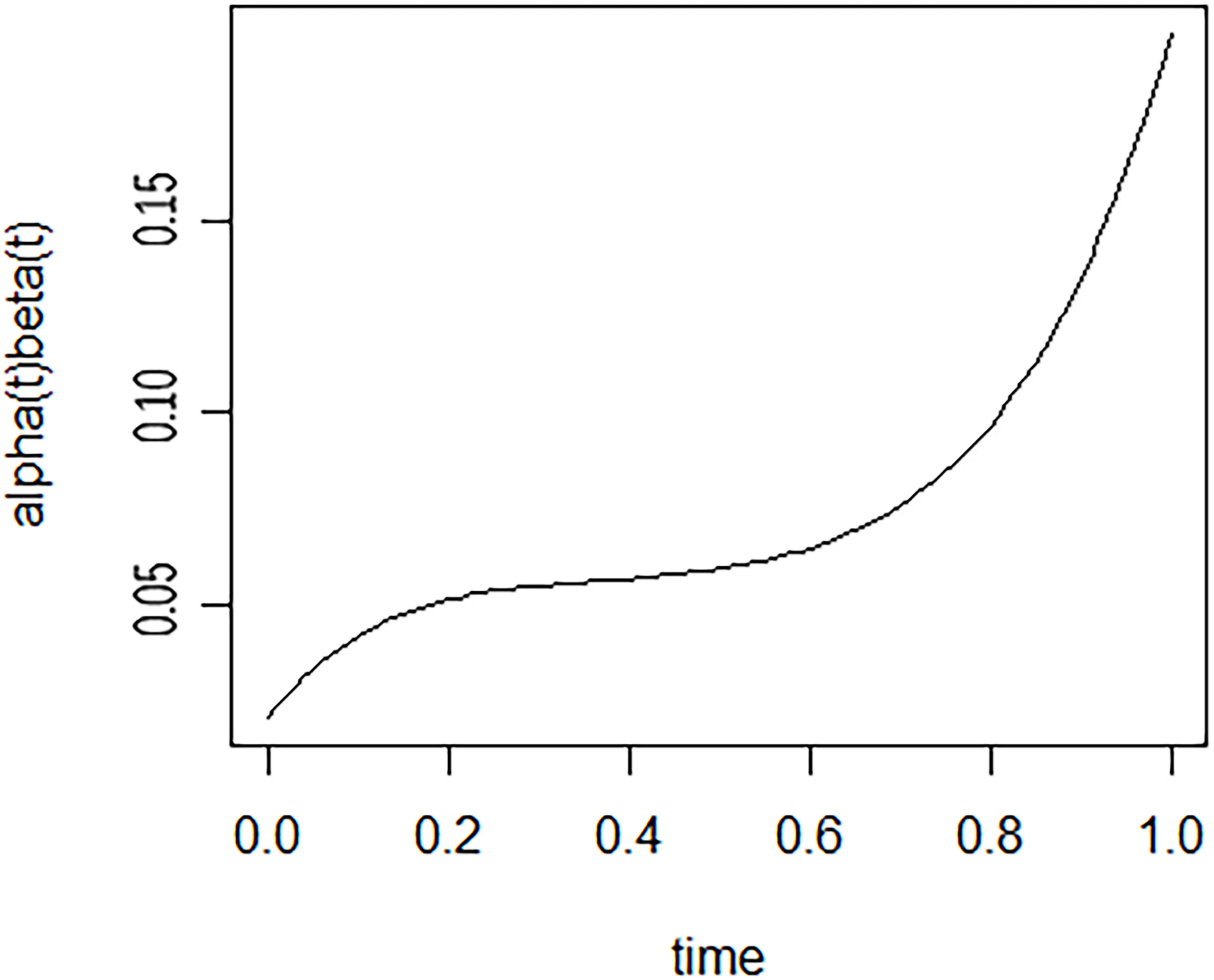
The dynamic mediating effect of depression between RBD and ADL.

After 10,000 iterations of the posterior parameter trajectory of the Bayesian dynamic mediation model, it is suggested that the model’s parameter convergence is reasonable (see **Supplementary Figure 2**). **Figure 2** showed the change in longitudinal mediation of depression between RBD and ADL. It was clear from the figure that the mediating effect of depression gradually increased over time.

### 3.3 Cognition as a mediator

Another Bayesian dynamic mediation model was constructed with RBD as the independent variable, ADL as the dependent variable, and cognition as the mediating variable. The indirect effects of cognition showed an increasing trend over time, increased from 0.062 to 0.136, a growth rate of approximately 119%. The standard deviations of the indirect effects were 0.021, 0.036, and 0.045, respectively, with a slight increase over time. At the time of 0.25, 0.50 and 0.75, the 95%*CI* of the indirect mediating effect was 0.038-0.086, 0.044-0.130 and 0.096-0.176, respectively (**Table 4**).

**Table 4:** Testing the mediating effect in a Bayesian mediation model with cognition as the mediating variable.

Time point	Indirect effect	Standard deviation	95%*CI*
0.25	0.062	0.021	(0.038, 0.086)
0.50	0.087	0.036	(0.044, 0.130)
0.75	0.136	0.045	(0.096, 0.176)

**Figure 3:**
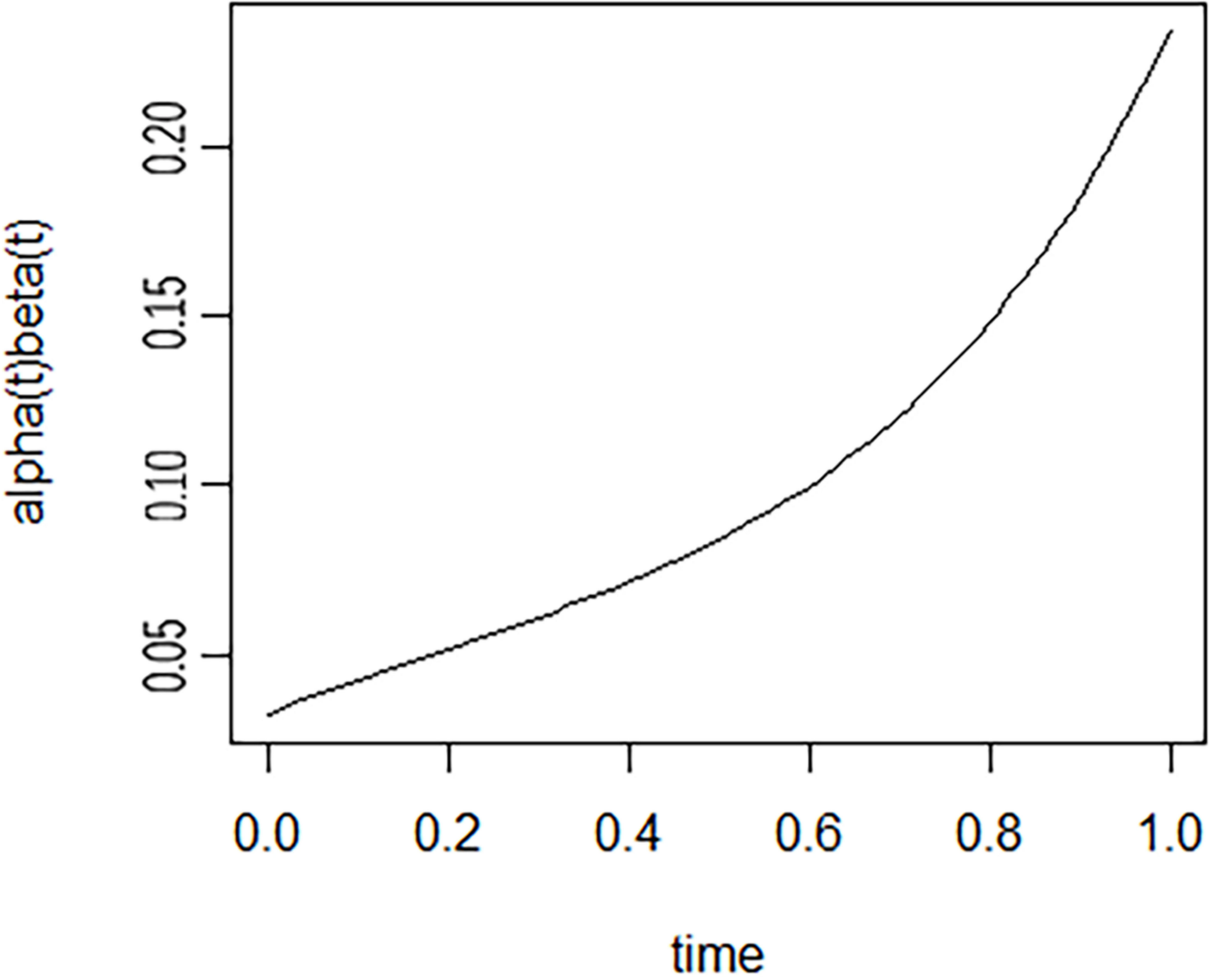
The dynamic mediating effect of cognition between RBD and ADL.

The posterior parameter trajectories of the Bayesian dynamic mediation model showed that the parameter convergence of the model is reasonable (see **Supplementary Figure 3**). **Figure 3** showed the longitudinal mediating change in cognition between RBD and ADL. It was clear from the graph that the indirect effects of cognition showed an increasing trend over time. Based on the mean of the indirect effects of depression or cognition (0.068, 0.095), the Monte Carlo simulation (2,000 times) showed that the statistical power of detecting the two indirect effects was 97.5% and 99.8%, respectively, and the power of detecting the total indirect effect was 99.9% at the current sample size (N = 337).

### 3.4 Depression and cognition as mediators

Using the bidirectional decomposition method for multiple time-varying mediation analysis (**Table 5**). The total effect of depression and cognition was 7.42, with a 95%*CI* of 4.48-10.36; the direct effect was 4.83, with a 95%*CI* of 2.13-7.53; and the indirect effect was 2.59, with a 95%*CI* of 1.43-3.75. The joint mediating effect of depression and cognition between RBD and ADL was significant. In the constructed multiple time-varying mediation model, the worsening symptoms of RBD led to an increase of 7.42 points in the ADL scores of patients with PD. Among these, RBD directly resulted in an increase of 4.83 points in ADL scores, accounting for 65.09%; RBD led to an increase of 2.59 points in the score of ADL through the combined mediating effect of depression and cognition, accounting for 34.91%. The residual between the TE and the total effect estimated by the model was 0.08, and the model had a very sufficient decomposition of the TE.

**Table 5:** Mediation effect test based on Multiple time-varying covariate mediation model.

Mediation effect		Effect value(%)	95%*CI*
Bidirectional decomposition method			
Total effect		7.42	(4.48, 10.36)
direct effect		4.83 (65.09)	(2.13, 7.53)
indirect effect		2.59 (34.91)	(1.43, 3.75)
*ξ*		0.08	(0.02, 0.14)
Path specification method			
Total effect		7.42	(4.48, 10.36)
direct effect		4.83 (65.09)	(2.13, 7.53)
indirect effect		2.59 (34.91)	(1.43, 3.75)
mediator variables	depression	1.37 (18.46)	(0.63, 2.11)
	cognition	1.22 (16.45)	(0.39, 2.05)

**Figure 4:**
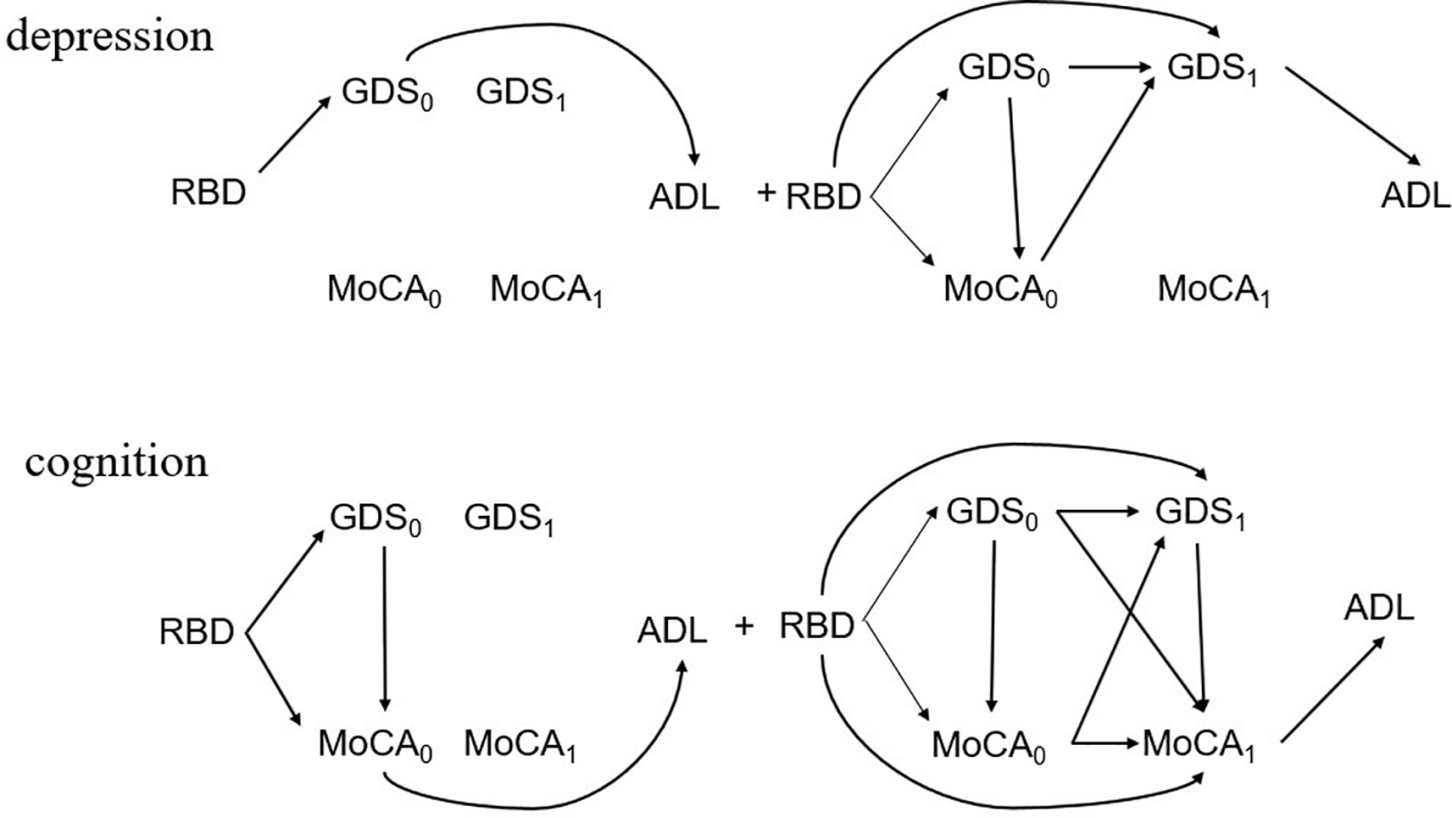
The mediation pathway diagram of depression or cognition at time *t* = 1 and 2.

Using the path specification method to further decompose the combined mediating effect of depression and cognition (**Table 5**). The indirect mediating effect value of depression was 1.37, accounting for 18.46%; the mediating effect value of cognition was 1.22, accounting for 16.45%. In the constructed multiple time-varying mediation model, the exacerbation of depression led to a decrease of 1.37 points in the score of ADL for PD patients; the decline in cognition led to a decrease of 1.22 points in the ADL score for PD patients. **Figure 4** shows the mediation pathway diagram to further illustrate depression or cognition’s path-specific effect at time *t* = 1 and 2.

## 4 Discussion

This study found that both depression and cognitive function in patients with PD had significant independent mediating effects and combined causal mediating effects between RBD and ADL. As the disease course prolongs, the mediating effects of depression and cognition on ADL showed continuous upward trend. This finding provided scientific basis for improving the self-care ability and overall quality of life of PD patients.

The total indirect effect was 99.9% at N = 337, which could be considered that the sample size meets the statistical needs. RBD exacerbated depressive symptoms, and deepening depression further compromised the ADL of PD patients. Liu et al. (23) indicated that, compared with PD patients without RBD, those with RBD exhibited a progressive increase in depressive severity over time. Subsequent analyses revealed that the causal mediating effect of depression on the RBD-ADL pathway strengthened longitudinally, indicating that the role of depression as a mediator became increasingly prominent. In parallel, RBD precipitated cognitive decline, which subsequently impairs ADL. The mediating effect of cognition also grew progressively over time. Consequently, a comparison of the magnitudes of the causal mediating effects exerted by depression and by cognition was warranted.

RBD can influence ADL through the combined mediating effects of depression and cognitive ability. Compared with PD patients without RBD, those with RBD shows more pronounced exacerbation of depressive severity and decline in cognitive function over time (24). Evidence suggests that PD patients with RBD exhibit more non-motor symptoms than those without RBD (25). Further decomposition of the combined mediating effects of depression and cognition reveals that the mediating effect of depression accounted for a larger proportion than that of cognition. In other words, RBD has more severe impact on ADL through depression than through cognition. The reasons for this phenomenon may be that depression can occur at any stage of the PD course (26), depressive symptoms are prone to oversight due to symptom overlap, and their core features like anhedonia, avolition may directly undermine the capacity for daily activities. Its onset often precedes non-motor symptoms, such as RBD, cognitive decline and so on. More importantly, the lack of specific diagnostic criteria for depression for PD has led to depression being undervalued in the clinical setting (27). Therefore, depression can lead to more severe functional disability, faster physical and cognitive degradation, higher mortality, poorer quality of life, and increased caregiver burden (28).

In the treatment of PD, the impact of non-motor symptoms should not be underestimated. RBD not only directly impairs patients’ daily functional performance but may also affect their ADL through the indirect effects of depression and cognitive function. Yin et al. (29) identified that an abnormal intracranial EEG, electromyographic coupling, during REM sleep serves as an electrophysiological marker of RBD in PD, disrupting this aberrant coupling through deep brain stimulation (DBS) could open a novel therapeutic avenue for RBD in PD. For PD patients with early-stage RBD, relevant health education can be provided; for patients with severe RBD symptoms, pharmacological treatments such as clonazepam and melatonin can be administered (30). Meanwhile, clinical emphasis on depression should be enhanced to prevent and improve cognitive function in PD patients, avoiding severe impacts on their ADL and quality of life.

Several limitations of this study should be acknowledged. Although the PPMI database is a large global cohort, patients mainly come from Europe and North America (31), and in the future we can use databases from other countries and regions for external validation. Sleep disorders include many symptoms, such as insomnia, excessive daytime sleepiness, restless legs syndrome, sleep breathing disorders and RBD, etc. These sleep disorders all have varying degrees of impact on ADL in PD patients, but this study only considers the factor of RBD. Sleep disturbances such as insomnia affect a majority of PD patients, with studies reporting prevalence rates between 27% and 80% (32), these conditions are also commonly present alongside RBD (4). In future research, we will further explore the impact of other sleep disorder manifestations on ADL.

## 5 Conclusions

In conclusion, this study constructed causal models and demonstrated that both depression and cognition independently and jointly causal mediating effects between RBD and ADL in PD patients. As disease duration increases, these mediating effects progressively strengthen, with the mediating effect of depression being relatively larger than that of cognition. Timely prevention of depressive mood and cognitive decline in the early stage of PD was particularly critical to reducing the harm caused by RBD, and could also play a positive role in improving the patients’ ADL and quality of life.

## Data Availability

The datasets presented in this study can be found in online repositories. The names of the repository/repositories and accession number(s) can be found below: https://www.ppmi-info.org/access-data-specimens/download-data, RRID : SCR_006431.
