# Supplementary material for: Depression and cognition mediated rapid eye movement sleep behavior disorder to improve the activities of daily living in Parkinson’s disease patients
Source: Front Psychiatry. 2026 Mar 20;17:1776297. doi: 10.3389/fpsyt.2026.1776297 (PMC13047128; doi:10.3389/fpsyt.2026.1776297)
Supplement: Supplementary file 1 [file SupplementaryFile1.docx]

Supplementary Material

# Supplementary Figures of Correlation analysis of RBDSQ, GDS-15, MoCA, UPDRS II. RBDSQ


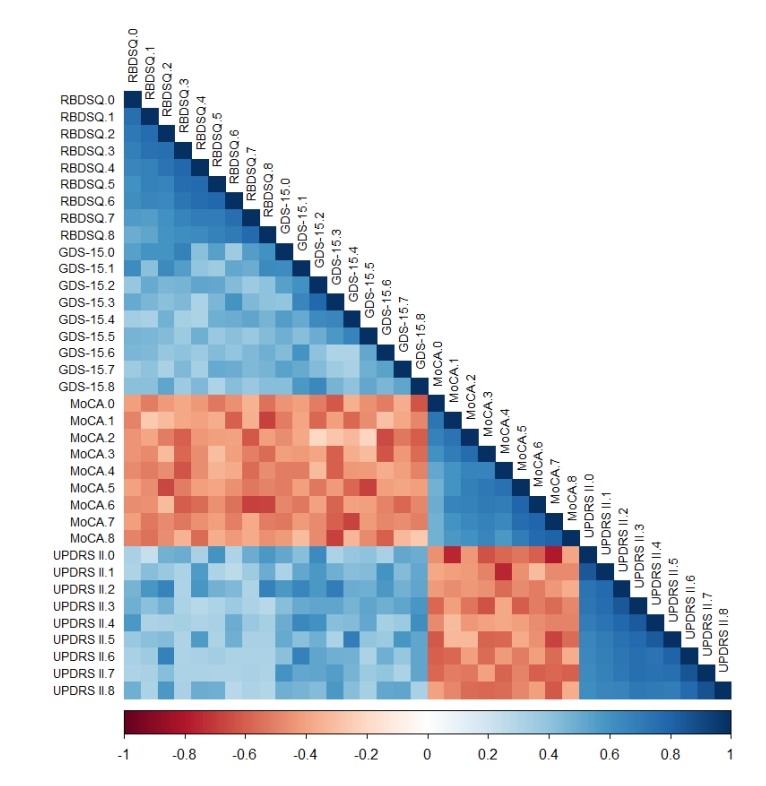


**Supplementary Figure 2.** Correlation analysis of RBDSQ, GDS-15, MoCA, UPDRS II. RBDSQ: REM Sleep Behavior Disorder Scale score, GDS-15: Short Depression Rating Scale score, MoCA: Montreal Cognitive Assessment Scale score, UPDRS II: Activities of Daily Living Scale score. 0, 1, 2, 3, 4, 5, 6, 7, 8: The baseline data and eight follow-up data

# Supplementary Figures of Bayesian Posterior Parameter Trace Plot


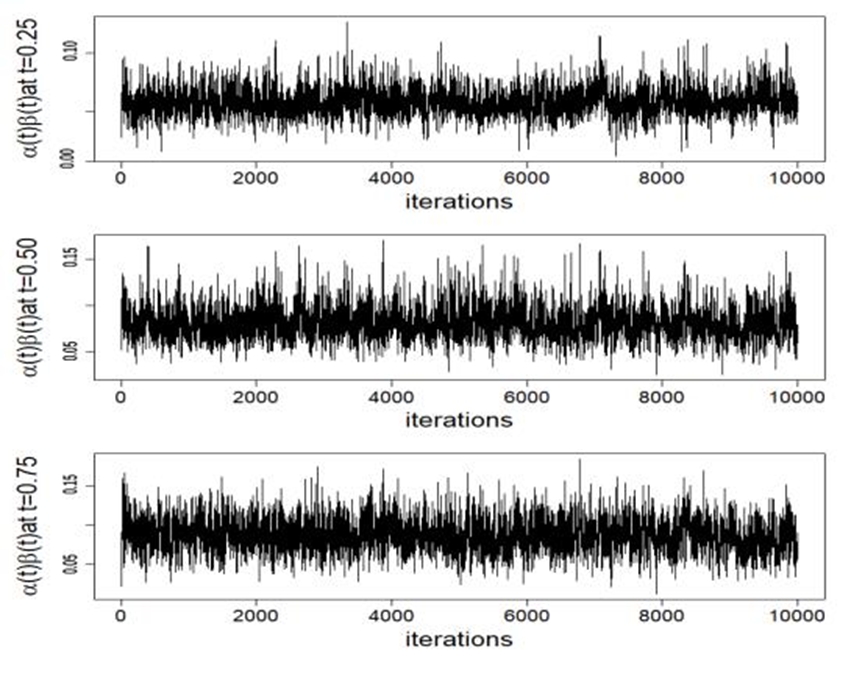


**Supplementary Figure 2.** Depression as the mediating variable


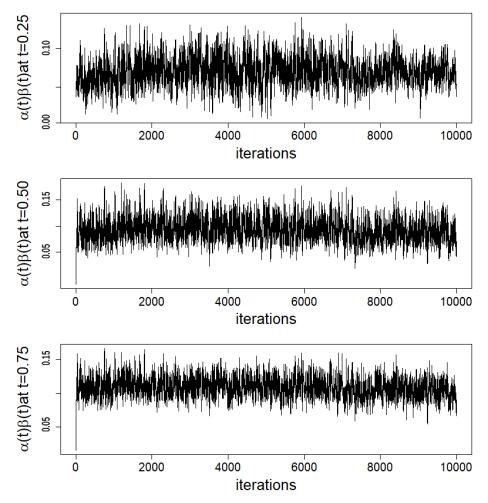


**Supplementary Figure 3.** Cognition as the mediating variable

# Bayesian Dynamic Mediation Analysis Code

exp1 <- read.table("exp1.csv", header = TRUE, sep = ",")

exp2 <- read.table("exp2.csv", header = TRUE, sep = ",")

###Create a function

DynMed<-function(data=NULL,timepoint =c(0.2,0.4,0.6,0.8),plot=TRUE){

nknot=9;nsim=10000;nburnin=5000

tmp.est=array(dim=c(length(timepoint),nsim))

est=array(dim=c(length(timepoint),nsim-nburnin))

library(MASS)

mydata=data; myK=nknot

my_t1<-mydata[,5]

my_t2<-my_t1^2

pre.k<-seq(0,1,length.out=myK+2)

myknot<-pre.k[2:(length(pre.k)-1)]

my_f<-function(x,knot){

myf<-NULL

for (i in 1:length(knot)){

myf[i]<-(ifelse(x>=knot[i],(x-knot[i])^2,0))

}

return(myf)

}

my_t3<-t(sapply(my_t1,my_f,knot=myknot))

my_v1<-as.numeric(mydata[,2])

my_m<-my_v4<-as.numeric(mydata[,3])

my_y<-as.numeric(mydata[,4])

my_v2<-my_v1*my_t1

my_v3<-my_v1*my_t2

my_v5<-my_v4*my_t1

my_v6<-my_v4*my_t2

my_v7<-my_v1*my_t3

my_v8<-my_v4*my_t3

N_obs<-as.numeric(as.vector(table(mydata[,1])))

N_subj<-as.numeric(length(unique(mydata[,1])))

sub<-as.numeric(match(mydata[,1], unique(mydata[,1])))

#################

alpha=array(dim=c(2*(3+myK),nsim))

alpha[,1]=rep(0,2*(3+myK));

beta=array(dim=c(3*(3+myK),nsim))

beta[,1]=rep(0,3*(3+myK));

tau_a=tau_b=tau_c=tau_d=tau_f=array(dim=c(1,nsim));

tau_a[1]=tau_b[1]=tau_c[1]=tau_d[1]=tau_f[1]=1;

tau_e2=tau_e3=array(dim=c(1,nsim));

tau_e2[1]=tau_e3[1]=1

tau_u1=tau_u2=array(dim=c(1,nsim));

tau_u1[1]=tau_u2[1]=1

u1=u2=array(dim=c(N_subj,nsim))

u1[,1]=rep(0,N_subj);

u2[,1]=rep(0,N_subj);

N_tot=sum(N_obs);

my_w1=cbind(my_t1,my_t2,my_t3,my_v1,my_v2,my_v3,my_v7);

my_w2=cbind(my_t1,my_t2,my_t3,my_v4,my_v5,my_v6,my_v8,my_v1,my_v

2,my_v3,my_v7);

#############################

my.hat<-function(x,alpha0,alpha1,alpha2,a1,a2,a3,a4,a5,a6,a7,a8,a9){

f1<-(ifelse(x>=0.1,(x-0.1)^2,0))

f2<-(ifelse(x>=0.2,(x-0.2)^2,0))

f3<-(ifelse(x>=0.3,(x-0.3)^2,0))

f4<-(ifelse(x>=0.4,(x-0.4)^2,0))

f5<-(ifelse(x>=0.5,(x-0.5)^2,0))

f6<-(ifelse(x>=0.6,(x-0.6)^2,0))

f7<-(ifelse(x>=0.7,(x-0.7)^2,0))

f8<-(ifelse(x>=0.8,(x-0.8)^2,0))

f9<-(ifelse(x>=0.9,(x-0.9)^2,0))

rr<-

alpha0+alpha1*x+alpha2*x^2+a1*f1+a2*f2+a3*f3+a4*f4+a5*f5+a6*f6+a7*f7 +a8*f8+a9*f9

return(rr)

}

############################

for (w in 2:nsim){

for (i in 1:N_subj){

sig_u1=(N_obs[i]*tau_e2[w-1]+tau_u1[w-1])^(-1);

sig_u2=(N_obs[i]*tau_e3[w-1]+tau_u2[w-1])^(-1);

temp_w1=my_w1[sub==i,];

temp_w2=my_w2[sub==i,];

mu_u1=sig_u1*(tau_e2[w-1]*sum(my_m[sub==i]-

temp_w1%*%alpha[2:(2*(3+myK)),w-1])+tau_u1[w-1]*alpha[1,w-1]);

mu_u2=sig_u2*(tau_e3[w-1]*sum(my_y[sub==i]-

temp_w2%*%beta[2:(3*(3+myK)),w-1])+tau_u2[w-1]*beta[1,w-1]);

u1[i,w]=rnorm(1,mu_u1,sqrt(sig_u1));

u2[i,w]=rnorm(1,mu_u2,sqrt(sig_u2));

}

sig_a0=(N_subj*tau_u1[w-1]+10^(-4))^(-1);

sig_b0=(N_subj*tau_u2[w-1]+10^(-4))^(-1);

mu_a0=sig_a0*tau_u1[w-1]*sum(u1[,w]);

mu_b0=sig_b0*tau_u2[w-1]*sum(u2[,w]);

alpha[1,w]= rnorm(1,mu_a0,sqrt(sig_a0));

beta[1,w]= rnorm(1,mu_b0,sqrt(sig_b0));

sig_a=diag(c(rep(1,2)*10^4,rep(1,myK)/tau_c[w-

1],rep(1,3)*10^4,rep(1,myK)/tau_a[w-1]));

temp_mu_a=array(dim=c(2*(3+myK)-

1,N_subj));temp_v_a=array(dim=c(2*(3+myK)-1,2*(3+myK)-1,N_subj));

for(i in 1:N_subj){

temp_w1=my_w1[sub==i,];

if(N_obs[i]==1){

temp_v_a[,,i]=(temp_w1)%*%t(temp_w1)*tau_e2[w-1];

temp_mu_a[,i]=(temp_w1)%*%t(my_m[sub==i]-u1[i,w])*tau_e2[w-1];

}else{

temp_v_a[,,i]=t(temp_w1)%*%diag(rep(1,N_obs[i]))%*%(temp_w1)*tau_e2[w

-1];

temp_mu_a[,i]=t(temp_w1)%*%diag(rep(1,N_obs[i]))%*%(my_m[sub==i]-

u1[i,w])*tau_e2[w-1];

}

}

sig_alpha=solve(apply(temp_v_a,c(1,2),sum)+solve(sig_a));

mu_alpha=sig_alpha%*%(apply(temp_mu_a,c(1),sum));

#sig_alpha=(sig_alpha+t(sig_alpha))/2;

alpha[2:(2*(3+myK)),w]=mvrnorm(1,mu_alpha,sig_alpha);

sig_b=diag(c(rep(1,2)*10^4,rep(1,myK)/tau_f[w-

1],rep(1,3)*10^4,rep(1,myK)/tau_b[w-1],rep(1,3)*10^4,rep(1,myK)/tau_d[w-

1]));

temp_mu_b=array(dim=c(3*(3+myK)-

1,N_subj));temp_v_b=array(dim=c(3*(3+myK)-1,3*(3+myK)-1,N_subj));

for(i in 1:N_subj){

temp_w2=my_w2[sub==i,];

if(N_obs[i]==1){

temp_v_b[,,i]=(temp_w2)%*%t(temp_w2)*tau_e3[w-1];

temp_mu_b[,i]=(temp_w2)%*%t(my_y[sub==i]-u2[i,w])*tau_e3[w-1];

}else{

temp_v_b[,,i]=t(temp_w2)%*%diag(rep(1,N_obs[i]))%*%temp_w2*tau_e3[w-

1];

temp_mu_b[,i]=t(temp_w2)%*%diag(rep(1,N_obs[i]))%*%(my_y[sub==i]-

u2[i,w])*tau_e3[w-1];

}

}

sig_beta=solve(apply(temp_v_b,c(1,2),sum)+solve(sig_b));

mu_beta=sig_beta%*%(apply(temp_mu_b,c(1),sum));

#sig_beta=(sig_beta+t(sig_beta))/2;

beta[2:(3*(3+myK)),w]=mvrnorm(1,mu_beta,sig_beta);

tau_a[w]=rgamma(1,10^(-3)+myK/2,scale=(10^(-

3)+sum(alpha[(3+myK+3+1):(2*(3+myK)),w]^2)/2)^(-1))

tau_b[w]=rgamma(1,10^(-3)+myK/2,scale=(10^(-

3)+sum( beta[(3+myK+3+1):(2*(3+myK)),w]^2)/2)^(-1));

tau_d[w]=rgamma(1,10^(-3)+myK/2,scale=(10^(-

3)+sum( beta[(2*(3+myK)+3+1):(3*(3+myK)),w]^2)/2)^(-1));

#intercept part

tau_c[w]=rgamma(1,10^(-3)+myK/2,scale=(10^(-

3)+sum(alpha[(3+1):(3+myK),w]^2)/2)^(-1))

tau_f[w]=rgamma(1,10^(-3)+myK/2,scale=(10^(-

3)+sum( beta[(3+1):(3+myK),w]^2)/2)^(-1))

res1_sum=0;res2_sum=0;

for(i in 1: N_subj){

temp_w1=my_w1[sub==i,];

temp_w2=my_w2[sub==i,];

tem_E=my_m[sub==i]-u1[i,w]-temp_w1%*%alpha[2:(2*(3+myK)),w];

tem_F=my_y[sub==i]-u2[i,w]-temp_w2%*% beta[2:(3*(3+myK)),w];

res1_sum=sum(tem_E^2)+res1_sum;

res2_sum=sum(tem_F^2)+res2_sum;

}

tau_e2[w]=rgamma(1,10^(-3)+(N_tot)/2, scale=(10^(-3)+res1_sum/2)^(-1));

tau_e3[w]=rgamma(1,10^(-3)+(N_tot)/2, scale=(10^(-3)+res2_sum/2)^(-1));

tau_u1[w]=rgamma(1,10^(-3)+(N_subj)/2, scale=(10^(-3)+sum((u1[,w]-

alpha[1,w])^2)/2)^(-1));

tau_u2[w]=rgamma(1,10^(-3)+(N_subj)/2, scale=(10^(-3)+sum((u2[,w]-

beta[1,w])^2)/2)^(-1));

aa=bb=NULL

aa=alpha[(3+myK+1):(2*(3+myK)),w]

bb= beta[(3+myK+1):(2*(3+myK)),w]

for( j in 1:length(timepoint)){

tmp.est[j,w]=my.hat(timepoint[j],

aa[1],aa[2],aa[3],aa[4],aa[5],aa[6],aa[7],aa[8],aa[9],aa[10],aa[11],aa[12])*my.ha

t(timepoint[j],

bb[1],bb[2],bb[3],bb[4],bb[5],bb[6],bb[7],bb[8],bb[9],bb[10],bb[11],bb[12]);

}

est = tmp.est[,((nburnin+1):nsim)]

}

output = cbind(timepoint,rowMeans(est), apply(est, 1, sd, na.rm = TRUE) ,

apply(est, 1, quantile, probs = c(0.025), na.rm = TRUE),apply(est, 1, quantile,

probs = c(0.975), na.rm = TRUE))

colnames(output) = c("t","mean", "sd", "95% CI", "95% CI")

print(output)

if(plot==TRUE){

tplot = seq(0,1,length=101)

aaplot = rowMeans(alpha[(3+myK+1):(2*(3+myK)),((nburnin+1):nsim)])

bbplot = rowMeans(beta[(3+myK+1):(2*(3+myK)),((nburnin+1):nsim)])

plot(tplot,

my.hat(tplot,aaplot[1],aaplot[2],aaplot[3],aaplot[4],aaplot[5],aaplot[6],aaplot[7],

aaplot[8],aaplot[9],aaplot[10],aaplot[11],aaplot[12])*my.hat(tplot,

bbplot[1],bbplot[2],bbplot[3],bbplot[4],bbplot[5],bbplot[6],bbplot[7],bbplot[8],

bbplot[9],bbplot[10],bbplot[11],bbplot[12]), "l", xlab="time",ylab =

"alpha(t)beta(t)")

invisible(tmp.est)

}

}

#Mediator 1

dmresult = DynMed(data = exp1, timepoint = c(0.25,0.5,0.75), plot=T)

# Mediator 2

dmresult = DynMed(data = exp2, timepoint = c(0.25,0.5,0.75), plot=T)

#Plot Bayesian Posterior Parameter Trace Plots

par(mfrow=c(3,1))

par(mar = c(4,5,2,5),oma =c(3,5,3,3),mgp=c(3,1,0))

plot(c(1:10000),dmresult[1,],"l",

ylab = expression(paste(alpha,"(t)",beta,"(t)","at t=0.25",sep="")),

xlab = "iterations",cex.lab=2,cex.axis=1.5)

plot(c(1:10000),dmresult[2,],"l",

ylab = expression(paste(alpha,"(t)",beta,"(t)","at t=0.50",sep="")),

xlab = "iterations",cex.lab=2,cex.axis=1.5)

plot(c(1:10000),dmresult[3,],"l",

ylab = expression(paste(alpha,"(t)",beta,"(t)","at t=0.75",sep="")),

xlab = "iterations",cex.lab=2,cex.axis=1.5)
